# Prevalence and factors related to dental caries among pre-school children of Saddar town, Karachi, Pakistan: a cross-sectional study

**DOI:** 10.1186/1472-6831-12-59

**Published:** 2012-12-27

**Authors:** Narendar Dawani, Nighat Nisar, Nazeer Khan, Shahbano Syed, Navara Tanweer

**Affiliations:** 1 Department of Community Dentistry, Dr. Ishrat Ul Ebad Khan Institute of Oral Health Sciences, DUHS, Rafiqui Shaheed Road, Karachi, Pakistan; 2 Department of Community Medicine, Dow University of Health Sciences, Baba-E-Urdu Road, Saddar, Karachi, Pakistan; 3 Department of Research, Dow University of Health Sciences, Baba-E-Urdu Road, Saddar, Karachi, Pakistan; 4 Dr. Ishrat Ul Ebad Khan Institute of Oral Health Sciences, DUHS, Rafiqui Shaheed Road, Karachi, Pakistan

**Keywords:** Dental caries, Prevalence, Pre-school children, Pakistan

## Abstract

**Background:**

Dental caries is highly prevalent and a significant public health problem among children throughout the world. Epidemiological data regarding prevalence of dental caries amongst Pakistani pre-school children is very limited. The objective of this study is to determine the frequency of dental caries among pre-school children of Saddar Town, Karachi, Pakistan and the factors related to caries.

**Methods:**

A cross-sectional study of 1000 preschool children was conducted in Saddar town, Karachi. Two-stage cluster sampling was used to select the sample. At first stage, eight clusters were selected randomly from total 11 clusters. In second stage, from the eight selected clusters, preschools were identified and children between 3- to 6-years age group were assessed for dental caries.

**Results:**

Caries prevalence was 51% with a mean dmft score being 2.08 (±2.97) of which decayed teeth constituted 1.95. The mean dmft of males was 2.3 (±3.08) and of females was 1.90 (±2.90). The mean dmft of 3, 4, 5 and 6- year olds was 1.65, 2.11, 2.16 and 3.11 respectively. A significant association was found between dental caries and following variables: age group of 4-years (p-value ² 0.029, RR = 1.248, 95% Bias corrected CI 0.029-0.437) and 5-years (p-value ² 0.009, RR = 1.545, 95% Bias corrected CI 0.047-0.739), presence of dental plaque (p-value ² 0.003, RR = 0.744, 95% Bias corrected CI (−0.433)-(−0.169)), poor oral hygiene (p-value ² 0.000, RR = 0.661, 95% Bias corrected CI (−0.532)-(−0.284)), as well as consumption of non-sweetened milk (p-value ² 0.049, RR = 1.232, 95% Bias corrected CI 0.061-0.367).

**Conclusion:**

Half of the preschoolers had dental caries coupled with a high prevalence of unmet dental treatment needs. Association between caries experience and age of child, consumption of non-sweetened milk, dental plaque and poor oral hygiene had been established.

## Background

Dental caries is highly prevalent among children and persists to be a significant public health problem worldwide [1]. It has detrimental consequences on children’s quality of life by inflicting pain, premature tooth-loss, malnutrition and finally influences overall growth and development [2]. The children suffering from poor oral health are 12 times more likely to have restricted activity days as compared to those who did not [3]. The prevalence of dental caries among pre-school children of developed nations has been declining over the past few decades. However, current evidence showed that this decline has ceased in certain developed countries [4,5], but the prevalence is still high among preschoolers of developing nations [6-8]. In India, findings of two studies showed prevalence of dental caries to be 51% and 54.1% respectively [9,10]. As for local statistics, Amynah et al [11] reported a caries experience of 29.1% with a mean dmft of 1.14 ±2.223 among preschoolers residing in Clifton region of Karachi city while a report from city of Lahore by Sufia et al [12] observed a caries prevalence of 40.1% with a mean dmft score of 1.85 ± 3.26 among 3-5-years old children.

Regarding the etiology of dental caries, four main factors have been identified; namely, i) bacteria, ii) fermentable carbohydrates, iii) a susceptible tooth surface, and iv) time [13,14].

Additionally, some socio-demographic and behavioral indicators that prone an individual to increased caries experience include: presence of plaque, poor oral hygiene, increasing age, gender, inadequate tooth-brushing habits, frequency and timing of consumption of sugar-containing drinks [15].

Dental caries is a preventable disease and if the burden of factors leading to such condition is known only then can better health education activities be planned. The identification of high-risk groups provides motivation to enhance community awareness and its involvement in preventive efforts; as well as re-orient oral health services towards oral health promotion and prevention [16].

There is scarcity of updated data about prevalence of dental caries among Pakistani pre-school children especially of the region under study. The reason for this neglect may be either the perception that primary teeth are not as important as the permanent counterparts or the inaccessibility and difficulty of examining such young children. An estimate of dental caries prevalence in Pakistani pre-school children would be beneficial to employ control as well as preventive measures at an early age of the child. This, in effect, would lead to an improved dental health status, retention of teeth for longer duration, and by large enable them to lead a good quality life.

Therefore, aim of the present study is; firstly to measure the frequency of dental caries among the least examined group (3-6-years old children) of Karachi, Pakistan and secondly, to identify the factors related to caries burden among children of identical age-bracket.

## Methods

A cross sectional study was conducted in Karachi, Pakistan. It is the largest city encompassing diverse inhabitants and comprises of 18 towns. According to USAID Country Health statistical report of 2009; the total preschool children population in Pakistan is estimated to be 22,476,931 [17] of which approximately 20.9% [18] attend the preprimary school. The current study was conducted in Saddar town, a densely populated town in the central part of city [19] constituting of total 11 union councils. A sample size of 957 was calculated through a computer software program Epi-Info 6. The prevalence of 44% [20] was taken as reference caries prevalence among five year old children with 5% margin of error, 95% Confidence Interval and 80% Power of test and it was rounded off to include 1000 participants. The sample was drawn using two-stage cluster sampling. In first stage, considering each union council as an individual cluster, eight clusters were randomly picked out of total 11 clusters; additionally the total number of preschools located in Saddar town was identified. In second stage, from eight selected clusters, respective preschools were identified and the required sample size of 1000 preschool children (who matched the inclusion criteria) was achieved from the selected pre-schools obtaining prior consent for conducting the research.

Three to six-years old children of both genders enrolled in selected preschools having deciduous dentition were included whereas those above the age of six having at least one permanent tooth or suffering from periodontal conditions or birth-defects were excluded from the research study. Hence, a total of one thousand 3-6-years old children attending kindergartens were interviewed to assess their oral hygiene and eating habits followed by a diagnostic examination for dental caries employing the universal dmft index [21]. The WHO criterion was used for diagnosing dental caries [22] while presence of dental plaque and assessment of oral hygiene was assessed solely through visual examination without employing any validated index since it was not the prime objective of the study. Caries severity was assessed via dmft index by categorizing the score of decayed component into i. Very mild (one tooth), ii. Mild (2–3 teeth), iii. Moderate (4–5 teeth) and iv. Severe (6+ teeth) [23]. Dental examination was done with the child either seated on an ordinary chair or in a knee to knee position depending on his/her behavior and age. An autoclavable sterilized mouth mirror and a CPI probe was used for the examination. The probe was used very cautiously to prevent damage to the sound intact enamel surface and the probe was used specifically to confirm the caries diagnosis. In case of any doubt the tooth was marked as sound. No radiographs were taken. Single dentist with over two years of experience conducted the clinical examination as well as the interview of every child for assessment of their dietary and oral hygiene habits. The chief examiner was calibrated against a standard examiner before initiating the research. To determine the reproducibility of diagnosis, 20 children were re-examined after a period of two weeks. Thereby, employing “Kappa” test for measuring percent agreement; the intra-examiner and inter-examiner percent agreement values of 93% and 90% were achieved.

Statistical Package for Social Sciences (SPSS) Version 20 was used to enter and analyze data. Descriptive data included frequencies, percentages, means, and standard deviations of study variables.

Conditional Univariate logistic regression analysis was used to depict statistically significant association of study variables (²0.2) with the outcome variable (dental caries). The findings were reported through p-value, Risk Ratio (RR), and 95% Confidence Interval (CI). The variables selected at the Univariate stage were adjusted for cluster effect by Bootstrap method which is based on estimation through re-sampling with replacement from the original sample to obtain adjusted p-value and 95% Bias corrected Confidence Interval (CI).

Significant variables according to p-value were further analyzed by Multivariate logistic regression to reduce confounders. The findings were reported through p-value, Risk Ratio (RR), and 95% Confidence Interval (CI). Again, adjusted p-value and 95% Bias corrected Confidence Interval (CI) were obtained after adjustment of cluster effect through Bootstrap method.

Ethical approval for the study was obtained from the Institutional Review Board of Dow University of Health Sciences. Furthermore, a written consent for participation in the study was obtained from the parents of respective children.

## Results

Out of 1000 pre-school children examined, 608 were girls and 392 boys with a mean age of 4.31 (±0.76). The mean age of males was 5.6 years and of females was 4.6 years respectively.

The overall caries prevalence in the study population was 51% with an overall mean dmft score of 2.08 (±2.97) of which decayed component comprised of 1.95 (±2.7), missing component 0.10 (±0.6) and filled component only 0.02 (±0.26) (see Table 1). The mean dmft of males was 2.3 (±3.08) and of females was 1.90 (±2.90). The mean dmft of 3, 4, 5 and 6 year olds was 1.65, 2.11, 2.16 and 3.11 respectively. Hence, the dmft score increased as the age advances.

**Table 1:** Distribution of variables regarding dental caries status and cumulative dmft

**VARIABLE**	**FREQUENCY (n = 1000)**	**PERCENT**	**MEAN ± SD**
**dmft (cumulative):**	509	50.9	2.08 ± 2.97
Decayed	504	50.4	1.95 ± 2.77
Missing	41	4.1	0.10 ± 0.61
Filled	10	1.0	0.02 ± 0.264
**dmft(Gender-wise distribution):**			
male:	392	39.2	2.3 ± 3.08
female:	608	60.8	1.90 ± 2.90
**dmft (Age-wise distribution):**			
3 yrs	165	16.7	1.65
4 yrs	381	38.1	2.11
5 yrs	437	43.7	2.16
6 yrs	17	1.7	3.11
**dmft of Caries positive cases:**			
	509	50.9	4.08
Decayed			3.83
Missing			0.19
Filled			0.04
**dmft of Caries positive cases (Gender-wise distribution):**			
Males:	212	41.6	4.32
Females:	297	58.4	3.9

Among the children with clinical caries, mean dmft score was 4.08 with dt 3.83, mt 0.19 and ft 0.04 respectively. The percentage of caries positive males was 41.6% and females was 58.4% while the mean dmft of caries positive males was 4.32 and of caries positive females was 3.9.

Out of 50.4% children who had clinically decayed teeth, 10.8% had one tooth involved (very mild), 16.8% had 2–3 teeth involved (mild), 9.8% had 4–5 teeth (moderate) whereas children who had more than 6 decayed teeth (severe) were only 13% (see Table 2). Dental plaque was clinically visible in 54% (540) of the children and children having poor oral hygiene comprised 20% (201) in study sample. Twelve percent (126) children stated that they consume betel nut (chalia) which could be demonstrated by the stains on their teeth.

**Table 2:** Severity of dental caries and status of oral health

**VARIABLE**	**FREQUENCY (n = 1000)**	**PERCENT**
**Dental Caries Status:**		
Caries positive	509	50.9
Caries negative	491	49.1
**decayed category for severity**:		
0 (sound)	496	49.6
1 (1 tooth involved)	108	10.8
2 (2–3 teeth involved)	168	16.8
3 (4–5 teeth involved)	98	9.8
4 (6+ teeth involved)	130	13.0
**Presence of Dental Plaque:**		
Yes	540	54
No	460	46
**Poor Oral Hygiene:**		
Yes	201	20.1
No	799	79.9
**Consumption of Betel nut (Chalia):**		
Yes	126	12.6
No	874	87.4

### Descriptive details of possible factors related to dental caries

Regarding oral hygiene practices of pre-school children; ninety one percent (911) used a brush to clean their teeth while only five percent (55) of them did not clean their teeth at all. More than 50% (539) children brushed their teeth only once a day and that too was mostly (808) in the morning time. Almost 87% (866) children used a tooth-paste; irrespective of presence or absence of fluoride content as well as the brand to clean their teeth while eight percent (80) did not use any kind of oral cleaning aid. Thirty three percent (335) children did not gargle after consuming meals.

Regarding dietary habits, majority of children consumed flavored sweetened milk (680) instead of non-sweetened milk whose intake was only 27% (276). About Eighty percent (808 and 797) children were habitual consumers of confectionaries and sweetened juices and syrups.

The outcome measure of current study was dental caries (positive/negative). No statistically significant difference [p-value ²0.098, RR = 1.107, 95% CI (−0.025)-0.229] was observed in the caries experience across gender. Age group of five-years [p-value ²0.046, RR = 1.313, 95% CI 1.005-1.716, Bias corrected CI 0.072-0.508] and six-years old [p-value ²0.046, RR = 1.829, 95% CI 1.011-3.307, Bias corrected CI 0.158-0.939] children was significantly associated with dental caries i.e. these age group children had a higher chance of suffering from tooth decay and as the age advances children become more prone to tooth decay. Children with dental plaque deposition had 53% more chances of suffering from dental caries [p-value ²0.001, RR = 1.531, 95% CI 1.277-1.835, Bias corrected CI 0.296-0.555] while those having poor oral hygiene [p-value ²0.001, RR = 0.585, 95% CI 0.484-0.707, Bias corrected CI (−0.653)-(−0.410)] were found to be 1.7 times more prone to dental caries (see Table 3). Whereas, use of a toothpaste showed an insignificant association (p-value ²0.125) with dental caries in initial conditional Univariate regression, however when cluster adjustment via random effect logistic regression was performed, it was observed that those not using a toothpaste had 80% higher chances of tooth decay [p-value ²0.019, RR = 1.205, 95% CI 0.019-0.331] (see Table 4). Similarly, children not consuming non-sweetened milk had 24% higher chance of tooth decay [p-value ²0.034, RR = 1.249, 95% CI 1.018-1.534, Bias corrected CI 0.068-0.393]. On the other hand, prior to cluster adjustment, no association (p-value ²0.073) amongst intake of flavored sweetened milk and tooth decay was observed but after cluster adjustment by random effects logistic regression analysis, children consuming flavored sweetened milk had 1.19 times more chances of dental decay than those who were not using flavored sweetened milk [p-value ²0.014, RR = 0.838, 95% CI (−0.330)-(−0.032)] (see Table 5). Whereas, consumption of confectionaries [p-value ²0.274, RR = 0.880, 95% CI 0.699-1.107, Bias corrected CI (−0.310)-0.030] as well as sweetened juices and syrups (p-value ²0.839, RR = 0.984, 95% CI (−0.193)-0.142) had no significant relationship with dental caries.

**Table 3:** Conditional univariate logistic regression with random effects logistic regression analysis of socio-demography and oral health status related to dental caries

**Variables**	**Conditional univariate logistic regression**			**Random effects logistic regression (bootstrap)**	
	***p-value***	***Risk ratio***	***95% Confidence interval***	***p-value***	***95% Confidence interval***
**Age category**					
3 years	Ref	-		-	-
4 years	0.256	1.174	0.891-1.547	0.145	(−0.046)-0.397
5 years	0.046	1.313	1.005-1.716	0.007	0.072-0.508
6 years	0.046	1.829	1.011-3.307	0.007	0.158-0.939
**Gender**					
Male	0.258	1.107	0.928-1.320	0.098	(−0.025)-0.229
Female	Ref			-	-
**Presence of Dental Plaque**					
Yes	Ref	-	-	-	-
No	0.000	1.531	1.277-1.835	0.001	0.296-0.555
**Poor Oral Hygiene**					
Yes	Ref	-	**-**	-	-
No	0.000	0.585	0.484-0.707	0.001	(−0.653)-(−0.410)

**Table 4:** Conditional univariate logistic regression with random effects logistic regression analysis of oral hygiene factors related to dental caries

**Variables**	**Conditional univariate logistic regression**			**Random effects logistic regression (Bootstrap)**	
	***p-value***	***Risk ratio***	***95% Confidence interval***	***p-value***	***95% Confidence interval***
**Use Toothbrush**					
Yes	Ref	-	-	-	-
No	0.375	1.140	0.853-1.522	0.187	(−0.082)-0.321
**Miswak used**					
Yes	Ref	-	-	-	-
No	0.501	0.677	0.218-2.107	0.141	(−0.727)-3.002
**Do not clean teeth at all**					
Yes	Ref	-	-	-	-
No	0.244	0.813	0.574-1.151	0.064	(−0.396)-(−0.002)
**Frequency of brushing**					
Nil	Ref	-	-	-	-
Once	0.342	0.833	0.571-1.215	0.153	(−0.406)-0.076
Twice	0.711	0.929	0.629-1.372	0.576	(−0.317)-0.189
Thrice	0.447	0.824	0.501-1.356	0.265	(−0.528)-0.107
Occasionally	0.339	0.770	0.450-1.317	0.161	(−0.634)-0.047
**Use Toothpaste**					
Yes	Ref	-	-	-	-
No	0.125	1.205	0.949-1.530	0.019	0.019-0.331
**No sort of dentifrice used**					
Yes	Ref	-	-	-	-
No	0.710	0.942	0.690-1.288	0.577	(−0.269)-0.169
**Gargles after meals**					
Yes	Ref	-	-	-	-
No	0.373	1.086	0.906-1.302	0.215	(−0.054)-0.207

**Table 5:** Conditional univariate logistic regression with random effects logistic regression analysis of dietary factors related to dental caries

**Variables**	**Conditional univariate logistic regression**			**Random effects logistic regression (Bootstrap)**	
	***p-value***	***Risk ratio***	***95% Confidence interval***	***p-value***	***95% Confidence interval***
**Consume Non-sweetened Milk**					
Yes	Ref	-	-	-	-
No	0.034	1.249	1.018-1.534	0.003	0.068-0.393
**Consumed Sweetened Flavored Milk**					
Yes	Ref	-	-	-	-
No	0.073	0.838	0.691-1.017	0.014	(−0.330)-(−0.032)
**Consume Sweet Juice & Syrup**					
Yes	Ref	-	-	-	-
No	0.884	0.984	0.792-1.222	0.839	(−0.193)-0.142
**Use Confectionaries**					
Yes	Ref	-	-	-	-
No	0.274	0.880	0.699-1.107	0.125	(−0.310)-0.030

In order to evaluate the contribution of such factors to the overall variance, whilst controlling the confounding factors, conditional multivariate logistic regression was applied utilizing the bootstrap method for cluster adjustment. Hence, in the final multivariate model; prior to cluster adjustment, no specific age category had any association with dental caries but following random effect logistic regression, children aged four-years [p-value ²0.029, RR = 1.248, 95% Bias corrected CI 0.029-0.437) and five-years [p-value ²0.009, RR = 1.545, 95% Bias corrected CI 0.047-0.739) were found to be affected more as compared to other age categories thereby re-affirming that as the age of child advances, chances of dental caries increment. Those consuming non-sweetened milk had 77% less chances [p-value ²0.049, RR = 1.232, 95% Bias corrected CI 0.061-0.367) of tooth decay than those who consume milk with added sugars. In addition, children who had plaque deposition on their teeth were 1.3 times [p-value ²0.003 RR = 0.744, 95% Bias corrected CI (−0.4333)-(−0.169)] more prone to acquiring dental decay compared to their counterparts and those suffering from poor oral hygiene were 1.51 times more prone to dental caries [p-value ²0.000, RR = 0.661, 95% Bias corrected CI (−0.532)-(−0.284)] (see Table 6).

**Table 6:** Conditional multivariate logistic regression with random effects logistic regression

**Variables**	**Conditional multivariate logistic regression**			**Random effects logistic regression (Bootstrap)**	
	***p-value***	***Risk ratio***	***95% Confidence interval***	***p-value***	***95% Confidence interval***
**Age category**					
3 years	Ref	-	-	-	-
4 years	0.106	1.248	0.954-1.635	0.029	0.029-0.437
5 years	0.163	1.545	0.838-2.849	0.009	0.047-0.739
**Poor oral hygiene**					
Yes	Ref	-	**-**	-	-
No	0.000	0.661	0.540-0.810	0.001	(−0.532)-(−0.284)
**Presence of Dental Plaque**					
Yes	Ref	**-**	-	-	-
No	0.003	0.744	0.614-0.902	0.001	(−0.433)-(−0.169)
**Non-sweetened Milk**					
Yes	Ref	-	-	-	-
No	0.049	1.232	1.001-1.516	0.008	0.061-0.367

## Discussion

The statistics of this study revealed that approximately half of 3-6-years old study population suffered from dental caries in their primary dentition, hence, it is justifiable to state that we are in line with WHO/FDI goals for 2000, i.e. 50% of 5-6-years old children should be caries free [16]. In spite of this fact, this percentage is significantly high keeping in perspective the biological consequences and financial burden of treating the disease in question in accordance to our current low-budget healthcare system. However, some local studies [11,12,24,25] have determined caries prevalence among local preschool children to be somewhat lesser compared to the current estimate, even though the disparity is trivial. Its likely rationale might be either the socio-economic differences of the study group or a difference in the nutrition and dietary habits amongst residents of the two provinces. Conversely, a study conducted in Islamabad [1], reported a high dmft score amongst primary dentate children but the study sample included children who were already suffering from caries and visited the hospital for treatment purpose.

More or less equivalent prevalence rates were reported from our bordering country India [9,10] probably due to similar socio-demographic, cultural, dietary and oral hygiene behavior patterns among children of defined age-group. However, these estimates are drastically inferior compared to the Arab World [6,26-30] as well as certain other developing nations [8,16,31-34] where a healthy proportion of children having deciduous dentition were carrying the burden of dental caries. On the contrary, pre-school children residing in the developed countries have lower caries prevalence [35-41]. The plausible explanation for such discrepancy can be inequality in economic conditions and resources, effective fluoridation policy, efficiency of healthcare system, availability and consumption of refined sugars, standard of oral health awareness among public, dietary and oral hygiene lifestyles, as well as motivational status of parents and children. The dmft value of sample population had similar universal trends as the above mentioned prevalence of dental caries [32,33,35,39,41,42].

Decayed teeth formed the major component of total dmft score, followed by missing and the least contribution was of filled teeth. Comparable proportions are evident in majority of studies [1,30,41]. The attributed explanation might be that majority of children do not undergo dental restorations primarily because of high treatment cost, lack of affordable dental services and false perceptions of parents regarding significance of retaining primary teeth, while those who undergo treatment prefer extraction rather than restorations.

The male children had a higher dmft value compared to females demonstrating that girls are more conscious about their diet, oral health and hygiene, but the diffe-rence was not significant, recent studies reported likewise [6,10,11,34,35,41]; perhaps due to the fact that at this young age, children are not self-motivated about their dental health and rely mostly on their parents for the maintenance of their oral hygiene. Couple of studies have; however, found a significant difference between dmft scores of preschool boys and girls [12,29].

Regarding the disease severity among caries positive children, majority had 1–3 teeth involved whereas less than thirteen percent individuals had six or more teeth affected by caries. This severity value is superior compared to an Australian study [23] and a valid explanation would be that their overall caries experience was low compared to present research outcome.

Most children maintained a good oral hygiene whereas plaque accumulation was observed in approximately half of them, which is substantially better compared to children of Saudi Arabia [26,27] and Laos [43] but inferior than Belgian preschool population [36].

The dmft scores of 3-6-years old children increment as the age-bracket advanced [10,29,36,41]. The feasible rationale of this caries advancement would be that as children grow older; their diet pattern alters from home-made nutritious food to unhealthy snacks and junk food easily available at school canteens, their parent’s involvement in tooth-brushing practices diminish, and also the duration of teeth being exposed to the oral environment prolongs. Presence of dental plaque and poor oral hygiene were observed to be significantly associated with caries prevalence and such negative associations were also established by numerous studies [15,26,44].

With regard to oral hygiene practices of children, only a minor proportion did not brush their teeth whereas only half of the participants brushed once a day in the morning time among which majority used a tooth-paste and two-thirds of them rinsed their mouth after meals. The aforementioned brushing practices are comparable to those reported from Saudi Arabia [27] and Belgium [36] but notably better than practices of Kosovo children [45]. Brushing once a day routine might be considered a general hygiene practice or a social norm and sufficient to maintain the kids’ oral hygiene among our population. Large number of children consumed sweetened drinks and confectionaries; this percentage is enormously high compared to Nigerian children [46], and this high rate of sweet consumption may be because the children feel emotionally pleased with sweet consumables.

The frequency of tooth-brushing was not significantly associated with dmft score and similar outcome was reported in a Kosovo study [45]. The explanation of failure to establish a correlation could be possibly due to a large proportion of study participants brushed their teeth only once a day and the responses were not equally distributed. This study observed that use of toothpaste did not have a significant impact on the caries experience despite its fluoride content which has anti-bacterial and remineralization properties [47,48]. Caries experience among children consuming flavored sweetened milk did not differ significantly and this observation is in contrast to various studies which have established caries association with sweetened liquids [15,45] as sugars can even diminish the protective effects of milk ingredients if added in it. Insignificant associations were also established with tooth-brushing practice, consumption of sweet juices and syrups as well as confectionaries. Consuming milk without any sugar additives had a positive (protective) impact on the caries experience of preschool children and such finding is probably due to the protective contents present in milk such as calcium, phosphates, casein, lactoferrins [49].

## Conclusion

Fifty one percent of pre-school population bears the burden of dental caries with a mean dmft score of 2.08. A high prevalence of unmet health care need still exists in our part of the world; reflected through a high ‘decayed teeth’ score in dmft index. Furthermore; age advancement, presence of dental plaque as well as poor oral hygiene has a significant impact on caries experience; also a protective role of non-sweetened milk against tooth decay has been established.

### Limitations

Since this was a cross-sectional study; therefore it cannot establish temporal associations and in this regard future longitudinal studies are suggested to establish causal associations for risk factors with dental caries. Additionally, true results might have been hampered due to incorrect reporting (reporting bias) by the young children as well as inability to interview the parents regarding their children’s oral behaviors.

### Future recommendations

1. Timely referral and restorative management of children suffering from dental caries would reduce the burden of disease.

2. A multisectoral approach involving the country’s health and education department, public and private schools, non-government agencies, dental community as well as pharmaceuticals related to dental field should be implicated to highlight the issue of tooth decay and its detrimental consequences on children’s quality of life among the general public and thereby, designing and implementing efficient mass deterrent and curative approaches.

## Abbreviations

dmft: Number of decayed, missing and filled primary teeth; dt: Decayed primary teeth; mt: Missing primary teeth; ft: Filled primary teeth; dft: Decayed and filled teeth; dfs: Decayed and filled surfaces; SiC: Significant caries index; WHO / FDI: World health organization / federation dentaire internationale; SES: Socio-economic status; p-value: Level of significance; SD: Standard deviation; RR: Risk ratio; CI: Confidence interval.

## Competing interests

The authors declare that they do not have any competing interests.

## Authors’ contributions

ND conceived the study, collected and analyzed the data and finally wrote manuscript. NN managed, helped in analysis of data and writing manuscript and editing of manuscript. SBS and NT assisted in collection of data, literature search and editing manuscript. NK supervised the project in addition to editing and help in writing manuscript. All authors read and approved the final manuscript.

## Pre-publication history

The pre-publication history for this paper can be accessed here:

http://www.biomedcentral.com/1472-6831/12/59/prepub
